# MULTIFACETED LINKS BETWEEN SOCIAL RELATIONSHIPS AND THE DISABLEMENT PROCESS IN LATER LIFE

**DOI:** 10.1093/geroni/igad104.1009

**Published:** 2023-12-21

**Authors:** Yuan Zhang

**Affiliations:** Columbia University, New York City, New York, United States

## Abstract

While a negative association between social relationships and physical limitations or disability has been reported persistently, the possible reciprocal relationships and pathways through which social relationships affect physical function and disability remain to be explored. This study used longitudinal data from the US Health and Retirement Study (HRS) and the National Social Life, Health, and Aging Project (NSHAP) to examine the multifaceted links between different dimensions of social relationships and physical limitations/disability among older adults in the US. We used Structural Equation Models to explore pathways between social relationships and physical limitations in the HRS and longitudinal residual change models to examine the effects of social relationships on changes in physical limitations between two waves in NSHAP. Results show that for men, greater social integration may lower subsequent physical limitations, whereas, for women, social support may prevent declines in physical function. For both genders, greater physical limitations and disability significantly lower subsequent social integration. This work tested the potential causal benefits of the social relationship hypothesis and selection processes and incorporated different aspects of social relationships. As such, our findings advance the explanations of the association between social relationships and later-life health and help better understand the determinants of the disablement process.

